# Complete Genome of *Clavibacter michiganensis* subsp. *sepedonicusis* Siphophage CN1A

**DOI:** 10.1128/genomeA.00865-13

**Published:** 2013-12-05

**Authors:** Rohit R. Kongari, Guichun W. Yao, Karthik R. Chamakura, Gabriel F. Kuty Everett

**Affiliations:** Center for Phage Technology, Texas A&M University, College Station, Texas, USAa; Department of Plant Pathology and Microbiology, Texas A&M University, College Station, Texas, USAb

## Abstract

*Clavibacter michiganensis* subsp. *sepedonicusis* is a Gram-positive actinomycete that is the causative agent of the potato disease ring rot. Here, we announce the complete genome sequence of the *Clavibacter michiganensis* subsp. *sepedonicusis* siphophage CN1A. CN1A is only the second fully sequenced *Clavibacter michiganensis* subsp. *sepedonicusis* phage reported to date. Core and unique features of its genome are described.

## GENOME ANNOUNCEMENT

*Clavibacter michiganensis* is an aerobic, nonsporulating, Gram-positive actinomycete that currently constitutes the only species within the genus *Clavibacter* (1). *Clavibacter michiganensis* subsp. *sepedonicusis* is a plant pathogen that is the causative agent of ring rot in potatoes (2, 3). Phage therapy presents an attractive option to the produce and farming industries for biocontrol of this pathogen. To date, there has been only one completely sequenced genome and one partially sequenced genome of *Clavibacter* phages, those of CMP1 (GenBank accession no. NC_013698.1) and CN77, respectively, both isolated against *Clavibacter michiganensis* subsp. *michiganensis*, a tomato pathogen (4).

Bacteriophage CN1A (kindly provided by A. Vidaver, University of Nebraska) was isolated based on its ability to grow on *Clavibacter michiganensis* subsp. *sepedonicusis*. Phage DNA was sequenced using 454 pyrosequencing at the Emory GRA Genome Center (Emory University, Atlanta, GA). Trimmed FLX Titanium reads were assembled to a single contig at 121-fold coverage using the Newbler assembler, version 2.5.3 (454 Life Sciences), at default settings. PCR confirmed completed contigs. Genes were predicted using GeneMarkS (5), and gene predictions were corrected using software tools available on the Center for Phage Technology (CPT) Portal (https://cpt.tamu.edu/cpt-software/portal/). Electron microscopy was performed at the Texas A&M Microscopy and Imaging Center.

CN1A is a siphophage with a unique genome of 55,837 bp. It has a G+C content of 62.1%, which is noticeably lower than the 72.6% G+C content of its host (3). The unit genome has a 90.1% coding density, with 78 predicted coding sequences and one tRNA gene. Based on BLASTp and InterProScan results, 18 genes were predicted to encode proteins with a known function (6, 7). Of the rest, 15 genes were hypothetical conserved genes and 45 were hypothetical novel genes. An examination of the raw sequence data using the PAUSE (https://cpt.tamu.edu/cpt-software/releases/pause/) method revealed that CN1A has a long terminal repeat of 952 bp.

The annotated genes correspond to core functions such as replication, nucleotide metabolism, morphogenesis, and lysis. Genes encoding replication proteins include those for helicase, DNA polymerase exonuclease subunit epsilon, and a single-stranded DNA (ssDNA) binding protein. A T4 PseT-like polynucleotide 5′-kinase/3′-phosphatase gene and a T7 gp*1*.*7*-like thymidylate kinase gene were also identified (8, 9). Genes for morphogenesis proteins encode a capsid protein, a large terminase, a tape measure protein, and two tail fiber proteins. The capsid protein was identified by its Big2 Ig-like domain (10). The tail proteins were identified by their SGNH hydrolase-type esterase domains (InterPro database entry IPR01381), which are often associated with phage tail fiber proteins (11, 12). The tape measure protein has two putative transmembrane domains, possibly suggesting a mechanism for phage DNA transport past the bacterial cytoplasmic membrane (13). Additionally, one GIY-YIG and two HNH homing endonucleases were found. The lysis cassette consists of a d-alanyl-d-alanine-carboxypeptidase adjacent to two transmembrane domain (TMD)-containing proteins (one with a single TMD and one with four TMDs). Presumably, these proteins are a holin/antiholin pair, although it is not yet known which protein belongs to which function.

### Nucleotide sequence accession number.

The genome sequence of phage CN1A was contributed to GenBank with the accession number KF669650.
